# A new species of *Lathrolestes* (Hymenoptera, Ichneumonidae) from Ecuadorian Amazonia, with a key to the Neotropical species of the genus

**DOI:** 10.3897/zookeys.251.3709

**Published:** 2012-12-18

**Authors:** Alexey Reshchikov, Anu Veijalainen, Ilari E. Sääksjärv

**Affiliations:** 1 The Insect Growth, Development and Behaviour Regulators Laboratory, Institute of Plant Protection, The Russian Academy of Agricultural Sciences, 3 Podbelsky shosse, Pushkin, St. Petersburg, Russia, 189620; 2 Zoological Museum, Section of Biodiversity and Environmental Sciences, Department of Biology, FIN-20014, University of Turku, Finland

**Keywords:** Amazonia, canopy, Ctenopelmatinae, parasitoid wasp, tropical rainforest, Yasuní

## Abstract

Here we describe and illustrate a new parasitoid wasp species, *Lathrolestes gauldi* **sp. n.** from the lowland rainforest of eastern Ecuador and provide a key to the Neotropical species of the genus. This is the first record of the subfamily Ctenopelmatinae from Ecuador.

## Introduction

The Ctenopelmatinae is a species-rich subfamily that includes mostly koinobiont endoparasitoids of sawfly larvae (Hymenoptera: Symphyta). The subfamily is expected to be more diverse in temperate than tropical zones as the primary host groups are relatively scarce in tropical rainforest habitats (Gauld et al. 1997, Veijalainen et al. 2012). In the Neotropics, the subfamily’s distribution extends across the entire region, but ctenopelmatines are still rarely encountered in Neotropical arthropod samples. The region’s ctenopelmatine fauna is very poorly known – there are only 69 described species from South America, Central America and Mexico (Cresson 1874, Townes 1970, Lanfranco 1980, Graf et al. 1991, Barron 1994, Gauld et al. 1997, Reshchikov 2011). In the most recent large study on Neotropical ctenopelmatines, 42 species from Costa Rica belonging to 14 genera were reported (Gauld et al. 1997).

In the present study, we describe a new species of *Lathrolestes* Foerster from Ecuadorian Amazonia. *Lathrolestes* is a large genus of the ctenopelmatine tribe Perilissini with 85 described species including 4 species from Costa Rica (Yu et al. 2012). The new species, *Lathrolestes gauldi* sp. n., was collected by canopy fogging in the lowland rainforests of Ecuadorian Amazonia. This represents the first record of the subfamily Ctenopelmatinae from Ecuador and the Neotropical rainforest canopy. We also provide a key to four species of *Lathrolestes* occurring in the Neotropical region and six closely related species from the southern United States and Mexico.

## Material and methods

The holotype specimen was collected by Dr. Terry L. Erwin and his research team from the canopy of a lowland *tierra firme* rainforest at Onkone Gare, Department of Orellana, Ecuador, on the 2nd of July, 1995. The site is located near the Yasuní National Park 216 m.a.s.l. in primary rainforest where the vegetation is old and diverse. The annual precipitation exceeds 2500 mm and the temperature always remains above 10 degrees Celsius. For a more specific description of the study site, see Lucky et al. 2002 and Erwin et al. 2005.

**Figure 1.**
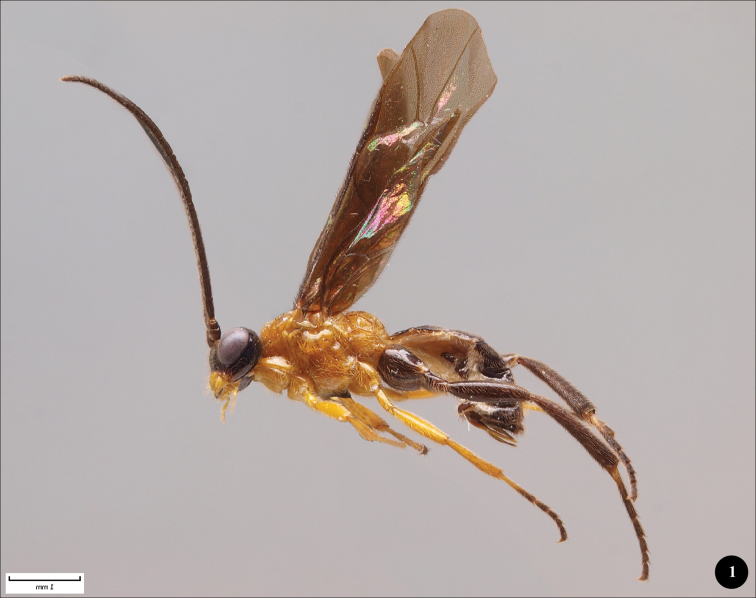
*Lathrolestes gauldi* sp. n.♀, holotype, habitus.

**Figures 2–5.**
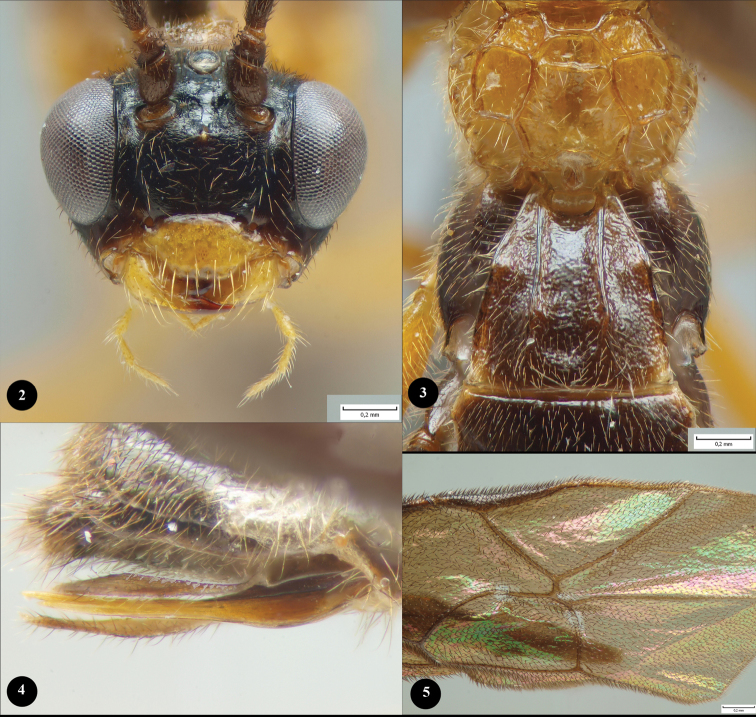
*Lathrolestes gauldi* sp. n. ♀, holotype. **2** face **3** propodeum and first metasomal tergite **4** ovipositor **5** areolet of the fore wing.

The lateral illustration (Fig. 1) of *Lathrolestes gauldi* shows the habitus and coloration of the species. In addition, we provide more detailed illustrations of the face (Fig. 2), propodeum (Fig. 3), ovipositor (Fig. 4) and areolet of the fore wing (Fig. 5) of the holotype specimen. All digital pictures were taken using an Olympus SZX16 stereomicroscope attached to an Olympus E520 digital camera and combined using the CombineZP program created by Alan Hadley (http://www.hadleyweb.pwp.blueyonder.co.uk/index.htm). The key below is modified from the one presented in Gauld et al. (1997). The morphological terminology follows mainly that of Gauld et al. (1997), wing vein nomenclature is based on Ross (1936).

## Results

### Taxonomy

Key to the Central and South American species of *Lathrolestes*

**Table:** 

1	Metasoma entirely black (Fig. 1). Wings strongly infuscate. Fore wing with resemblance of areolet (Fig. 5). First metasomal tergite short, almost as wide as long. Area superomedia twice as wide as long (Fig. 3). Ecuador	*Lathrolestes gauldi* sp. n.
–	Metasoma with yellow coloration or black with base of first tergite yellowish. Wings not infuscate or only slightly infuscate. Fore wing with areolet. First metasomal tergite longer, 1.25–2 times as long as wide. Area superomedia elongate	2
2	Mesoscutum with notaulus strongly impressed anteriorly; upper part of head and mesosoma granulate, matt; first tergite of metasoma anteriorly slender with lateromedian longitudinal carinae separated by about the diameter of the spiracle. Costa Rica	*Lathrolestes karenae* Gauld, 1997
–	Mesoscutum with notaulus vestigial; upper part of head and mesosoma fairly smooth and polished; first metasomal tergite anteriorly moderately stout with lateromedian longitudinal carinae separated by far more than the diameter of the spiracle	3
3	Tergites of metasoma uniformly reddish-yellow	4
–	Tergites of metasoma black and yellow or only some tergites reddish-yellow	7
4	Fore wing hyaline at apex. First tergite of metasoma short, 1.25 times as long as wide	5
–	Fore wing infuscate at apex. First tergite of metasoma longer, 1.6–1.7 times as long as wide	6
5	Area superomedia 1.4 times as long as wide. Costula arises from lateromedian longitudinal carina at the middle of area superomedia. White ring of antenna wide, approximately at flagellomeres 11–27. USA	*Lathrolestes asperatus* Barron, 1994
–	Area superomedia 1.8 times as long as wide. Costula arises from lateromedian longitudinal carina at upper part of area superomedia. White ring of antenna narrower, approximately at flagellomeres 11–18. Mexico	*Lathrolestes tepeyollotlis* Reshchikov, 2011
6	Area superomedia twice as long as wide. Costula absent. First tergite of metasoma without lateromedian longitudinal carinae. USA	*Lathrolestes erugatus* Barron, 1994
–	Area superomedia as long as wide. Costula present. First tergite of metasoma with lateromedian longitudinal carinae. Mexico	*Lathrolestes quetzalcoatlus* Reshchikov, 2011
7	Antennal flagellum orange-brown basally. Pterostigma translucent golden. Area superomedia 1.5 times as long as wide. Costa Rica	*Lathrolestes haroldi* Gauld, 1997
–	Antennal flagellum black basally. Pterostigma dark brown or black. Area superomedia shorter, 1.1–1.4 times as long as wide or longer, 1.6 times as long as wide	8
8	Middle of face black. Area superomedia 1.6 times as long as wide. Posterior transverse carina straight. First tergite of metasoma twice as long as wide, with weak lateromedian longitudinal carina. Mexico	*Lathrolestes kukulcanis* Reshchikov, 2011
–	Face entirely yellow. Area superomedia 1.1–1.4 times as long as wide. Posterior transverse carina curved. First tergite of metasoma 1.6–1.9 times as long as wide with lateromedian longitudinal carina defined	9
9	Propodeum with posterior transverse carina formicate (curved towards metasoma). Area superomedia 1.1 times as long as wide. Mexico	*Lathrolestes xochiquetzalis* Reshchikov, 2011
–	Propodeum with posterior transverse carina concave or straight	10
10	Propleuron yellow. First tergite of metasoma with weak lateromedian longitudinal carinae. Propodeum with posterior transverse carina straight. Hind coxa and femur entirely yellow. Costa Rica	*Lathrolestes irenea* Gauld, 1997
–	Propleuron black. First tergite of metasoma with strong lateromedian longitudinal carinae. Propodeum with posterior transverse carina concave. Hind coxa yellow, ventrally black, hind femur yellow, externally black. Costa Rica	*Lathrolestes jennyae* Gauld, 1997

### Description

#### Lathrolestes gauldi sp. n.

urn:lsid:zoobank.org:act:9887C02B-B24F-415A-9E02-8D8254148620

http://species-id.net/wiki/Lathrolestes_gauldi

Figs 1 –5

##### Material examined.

Holotype female: **Ecuador**, Department of Orellana, Onkone Gare (00°39'25.7"S, 76°27'10.8"W), 2.vii.1995, T.L. Erwin (NMNH, Smithsonian institution).

##### Diagnosis.

This species differs from other speciesof the genusby the following character states: an entirely black metasoma and hind legs, dark wings, short first metasomal tergite, and short area superomedia (half as long as wide (Fig. 3)).

##### Description.

Female. Body length 8.0 mm, pubescent with white hairs. Antenna with 22 flagellomeres. Scape 0.54 times as long as wide. Head narrowed behind eyes, polished. Maximal length of temple equal to transverse eye diameter; minimum length of temple 0.67× transverse eye diameter. Face 1.08× height of eye; convex centrally. Clypeus separated from face by groove; at apex projecting strongly anteriorly; apical margin of clypeus moderately obtuse, with line of deep punctures. Clypeal foveae small, placed in impressions. Malar space 0.7× as long as basal mandible width. Occipital carina dorsally not broadly interrupted. Lower mandible tooth longer and narrower than upper.

Mesosoma smooth, polished, without punctures. Notaulus shallowly impressed at base. Epicnemial carina high. Hind tibia compressed. Claws elongate, not pectinate. Hind tarsus as long as hind tibia. Vein 3rs-m vestigial (Fig. 5). Second recurrent vein with a single bulla. Nervulus strongly postfurcal. Hind wing with nervellus intercepted below middle. Propodeal carinae complete, strongly raised; area superomedia half as long as wide (Fig. 3).

Metasoma compressed apically, polished, sparsely pubescent. First metasomal tergite 0.86× as long as apically wide; without shallow median longitudinal impression; with lateromedian longitudinal carinae, slightly curved at spiracles; with slightly enlarged epipleurae (Fig. 3). Second metasomal tergite transverse. Metasomal sternites small, sclerotized. Subgenital plate slightly notched at apical margin. Ovipositor straight, thin, stout at base, slightly up-curved, approximately as long as metasomal height, without notch (Fig. 4).

Coloration. Female. Head black. Clypeus and mandibles orange (Fig. 2). Mesosoma, fore and middle legs (except of apical part of middle tarsus) orange (Fig. 1). Hind legs and apical part of middle tarsus, metasoma black. Wings infuscate.

##### Host.

unknown.

##### Distribution.

Ecuador.

##### Etymology.

The new species is dedicated to the late Dr. Ian D. Gauld.

## Discussion

Only one specimen of *Lathrolestes gauldi* sp. n. has been found despite the large sampling effort taking place in many Amazonian study localities (see Veijalainen et al. 2012). However, this is a normal situation with rainforest ichneumonids which are relatively difficult to sample, even by using long-term sampling programs (Sääksjärvi et al. 2004). Thus, *Lathrolestes gauldi* sp. n. seems to be a rare species, and further sampling is needed to clarify its distribution in the Western Amazonia.

## Supplementary Material

XML Treatment for Lathrolestes gauldi
